# A Nanosheet-Assembled SnO_2_-Integrated Anode

**DOI:** 10.3390/molecules26206108

**Published:** 2021-10-10

**Authors:** Xiaoli Wang, Xinyu Zhao, Yin Wang

**Affiliations:** 1 Liaoning Key Laboratory of Chemical Additive Synthesis and Separation, Department of Chemistry and Environment Engineering, Yingkou Institute of Technology, Yingkou 115014, China; wangxldq@sina.com; 2 Inner Mongolia Key Laboratory of Carbon Nanomaterials, College of Chemistry and Materials Science, Inner Mongolia Minzu University, Tongliao 028000, China

**Keywords:** anode, flexible electronics, nanosheets, SnO_2_

## Abstract

There is an ever-increasing trend toward bendable and high-energy-density electrochemical storage devices with high strength to fulfil the rapid development of flexible electronics, but they remain a great challenge to be realised by the traditional slurry-casting fabrication processes. To overcome these issues, herein, a facile strategy was proposed to design integrating an electrode with flexible, high capacity, and high tensile strength nanosheets with interconnected copper micro-fibre as a collector, loaded with a novel hierarchical SnO_2_ nanoarchitecture, which were assembled into core–shell architecture, with a 1D micro-fibre core and 2D nanosheets shell. When applied as anode materials for LIBs, the resultant novel electrode delivers a large reversible specific capacity of 637.2 mAh g^−1^ at a high rate of 1C. Such superior capacity may benefit from rational design based on structural engineering to boost synergistic effects of the integrated electrode. The outer shell with the ultrathin 2D nanoarchitecture blocks can provide favourable Li^+^ lateral intercalation lengths and more beneficial transport routes for electrolyte ions, with sufficient void space among the nanosheets to buffer the volume expansion. Furthermore, the interconnected 1D micro-fibre core with outstanding metallic conductivity can offer an efficient electron transport pathway along axial orientation to shorten electron transport. More importantly, the metal’s remarkable flexibility and high tensile strength provide the hybrid integrated electrode with strong bending and stretchability relative to sintered carbon or graphene hosts. The presented strategy demonstrates that this rational nanoarchitecture design based on integrated engineering is an effective route to maintain the structural stability of electrodes in flexible LIBs.

## 1. Introduction

Nowadays, an urgent and key task for energy conversion storage systems, in particular lithium-ion batteries (LIBs), is to develop advanced electrode materials with mechanical durability and superior Li-storage performance for booming flexible energy storage applications in foldable smartphones, wearable electronic systems, and implantable device [1,2,3,4,5]. However, the design and fabrication of such a high-performance flexible electrode is still a major challenge via a facile method because of the lack of optimal materials with the feature of high special capacity and robust mechanical flexibility in electrochemical environments. The traditional slurry-coating fabrication technology is not suitable for flexible LIBs because the active materials often suffer from exfoliation or cracking in the process of frequent bending. Furthermore, these additional binders in slurry would hinder electronic transport and reduce the specific energy density of the battery, and conductive carbon black should be added into the slurry to improve electrical performance.

In order to keep pace with the development of flexible energy storage systems and solve these problems of excessive consumption of adhesives and carbon black (~30 wt%), one of the most effective strategies is to construct an integrated electrode instead of slurry-coating fabrication technology [6,7,8,9,10,11]. In this regard, searching for an appropriate flexible current collector and high-energy-density active material is crucial to achieving these goals. To date, noteworthy progress in novel flexible electrodes has been achieved through chemical synthesis routes, including hydrothermal [12], sol–gel techniques [13], and CVD [14]. Nevertheless, most reported flexible energy storage devices are performed by using bio-driven carbon or free-standing film, which are not strong enough to withstand frequent mechanical deformations during practical use. Hence, an integrated electrode with robust tensile strength and enhanced electrochemical performance is still plagued and needs to be further improved.

Moreover, group IV element Sn in the form of metal oxide (SnO_2_) has particularly attracted extensive attention as a promising anode material to replace conventional graphitic carbon in current LIBs because of its uniqueness in terms of low cost, safe working potential, high theoretical capacity, and environmental friendliness [15,16,17,18,19]. Nevertheless, the simple structure, relatively low intrinsic conductivity, and vast structural variation during the reversible insertion/deinsertion processes of the bulk SnO_2_ powder keep it from achieving its full capacitance potential. One of the effective strategies to mitigate these problems is to develop structural engineering, including morphology control and hybrid construction, which could alleviate the mechanical stress induced by large volume change and prevent aggregation of the active domains [20,21,22,23,24,25]. However, these materials still need to be mixed with a binder and carbon black and pressed onto metal substrates or, alternatively, by being deposited onto a conductive substrate before they are assembled into batteries, which makes them less flexible and have a low energy density. Integrated electrodes, in which electrochemically active nanostructures are conformably coated on conductive collectors, have been demonstrated with ultrafast power rate and long lifespan. The successful integration of the sturdy conductive matrix support with elegant nanostructures improves the electrode performance and endows it with robust mechanical flexibility. All these merits render this type of electrode very attractive for flexible power sources.

To combine the aforementioned merits and promote the development of flexible LIBs, herein, we devised a novel route to fabricate SnO_2_-integrated electrode assembled by core–shell architecture, with a 1D micro-fibre core and 2D nanosheets shell, targeting high capacity and tensile strength LIBs. The copper micro-fibre clothes function as a superior conductive pathway facilitating fast electrons transfer along the axial orientation but also provide a high mechanical substrate assisting independent growth of SnO_2_ nanosheets. The nanosheets shells are vertically distributed on copper micro-fibre, forming core–shell structure, which can provide a large electrode/electrolyte interfacial contact area. As expected, when measured as anode materials of LIBs, we obtained a reversible lithium storage capacity of 637.2 mAh g^−1^ at a current density of 1C. The presented synthetic strategy is effective and with low cost, which provides a novel route to design advanced electrode materials for flexible LIBs.

## 2. Materials and Methods

### 2.1. Sample Synthesis

Prior to the synthesis, a piece of copper micro-fibre (CMF) textile (approximately 5 × 5 cm^2^) (Liaoning Copper Group, Liaoyang, China) was treated by ultrasonication with 1 M hydrochloric acid solution (Aladdin, Shanghai, China), in order to remove the CuO layer. In a typical hydrothermal synthesis of the SnO_2_ nanosheets array, 12 mmol SnCl_2_•2H_2_O and 24 mmol NH_4_F were first mixed in 70 mL deionised water under magnetic stirring (Aladdin, China). The clear and transparent solution was continuously stirred for 0.5 h in the air. Then, the mixed solution was transferred into a 100 mL Teflon-lined stainless steel autoclave. Afterwards, the CMF substrate was immersed in the solution. The sealed autoclave was heated to 180 °C for 24 h. Subsequently, the grey cloth was cleaned repeatedly with deionised water and ethanol under ultrasonic treatment and then dried under N_2_ gas flow. To investigate the formation of the nanosheet SnO_2_, a series of parallel experiments were carried out by adjusting the molar ratio of fluoride/Sn.

### 2.2. Materials Characterisation

The crystallographic information of the as-prepared materials was recorded with powder X-ray diffractometry (XRD, Bruker D8 Advance, Bremen, Germany). The morphological features and microstructure of the sample were observed by using a field-emission scanning electron microscopy (FE-SEM, S-4800, Hitachi, Tokyo, Japan) and transmission electron microscopy (TEM, JEOL JEM-2100F, Japan). X-ray photoelectron spectroscopic (XPS, ESCALAB 250, Thermo Scientific, Waltham, MA, USA) was used to characterise the surface composition of the sample.

### 2.3. Electrochemical Measurements

Electrochemical properties were measured using coin cells (CR2025), which were assembled in an argon-filled glovebox (Mbraun, Unilab, Germany). The copper micro-fibre-textile-supported ultrathin SnO_2_ nanosheets were used directly as the working electrode for the subsequent electrochemical tests without binders and conductivity carbon black. The coated cloth was cut into disk electrodes (12 mm in diameter). To fabricate the SnO_2_ power working electrode, the active materials, super-P, and PVDF with a mass ratio of 80:10:10 were ground in NMP solvent to form a homogeneous slurry, which was then coated onto the Cu foil by the doctor blade method and dried by heating in a vacuum oven (Yiheng, Shanghai, China). The lithium foil was used as the cathode electrode. The commercial electrolyte in the present measurements was a mixture of LiPF_6_ in ethylene carbonate, dimethyl carbonate, and diethyl carbonate (EC-DEC-EMC, 1:1:1 in *v*/*v*). Galvanostatic cycling performances of the as-prepared coin cell were operated at room temperature on a multi-channel battery testing system (Land CT2001A, Wuhan, China) with a cut-off voltage of 1.2–0.01 V versus Li/Li^+^. Cyclic voltammetry (CV) curves were carried out by applying a CHI-760E electrochemical workstation at a scanning rate of 0.1 mV s^−1^.

## 3. Results and Discussion

**Figure 1:**
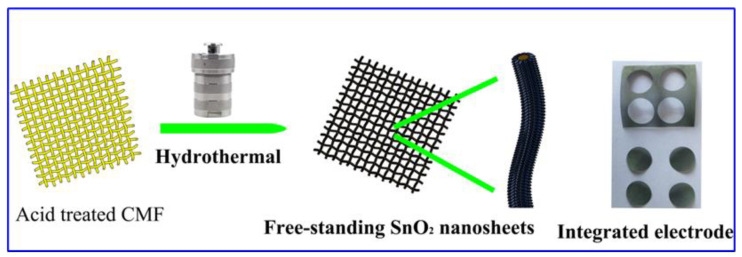
A schematic diagram for the fabrication procedure of SnO_2_ nanosheets on the CMF.

The flexible integrated electrode samples were fabricated via a facile in situ hydrothermal depositions of active material on the CMF without any post-treatment and use of conventional carbon black additive and binder. The overall fabrication process of the SnO_2_-integrated electrode is shown schematically in Figure 1. As detailed in the Experimental Section, the acid-treated copper micro-fibre textile was immersed in a mixed solution of stannous chloride dihydrate and ammonium fluoride, followed by a hydrothermal reaction at 180 °C for 24 h, yielding the large-size free-standing SnO_2_ nanosheets on the CMF. The SnO_2_-integrated electrode materials display superior flexibility and robust mechanical tensile, which can be cut directly into electrode pieces with a diameter of 12 mm.

**Figure 2:**
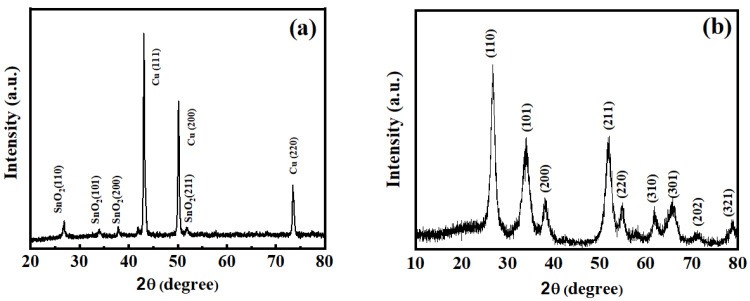
XRD pattern of SnO_2_: (**a**) nanosheets on the CMF; (**b**) nanosheets after HNO_3_ treatment.

Firstly, the powder X-ray diffraction (XRD) technique was provided to determine the composition characterisation of the as-prepared SnO_2_ samples. Figure 2a displays the crystallinity and phase purity of the SnO_2_ nanosheets assembled integration materials. The main three peaks at around 43.3, 50.4, and 74.1° correspond to the metal copper substrate. Removing the Cu element signals from the substrates, all the diffraction peaks can be well indexed to the tetragonal rutile structure of SnO_2_ (JCPDS Card no.41-1445). No impurities, such as SnO or Sn, were detected, indicating the formation of pure SnO_2_ nanosheets. In addition, a piece of SnO_2_/CMF-integrated composite sample was put into a concentrated nitric acid solution to remove the Cu substrates, and the resulting powers were purified with deionised water and ethanol for subsequent testing. As shown in Figure 2b, the XRD pattern indicated that the as-prepared powers after concentrated nitric acid treatment were in a pure tetragonal rutile crystalline phase. Supplementary Figure S1 presents the XRD pattern of the SnO_2_ nanoflower assembled nanosheets synthesised by hydrothermal method without CMF, which is in accordance with the above-mentioned sample.

**Figure 3:**
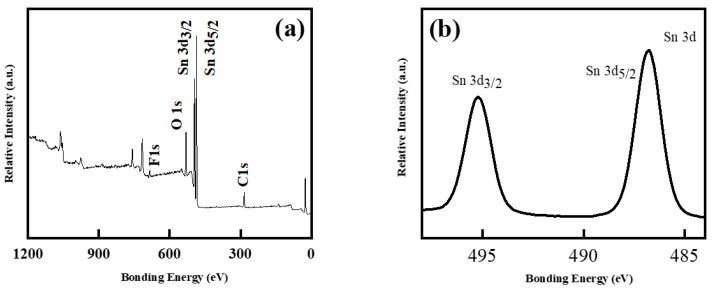
XPS spectrum of SnO_2_ nanosheets: (**a**) survey XPS pattern; (**b**) high-resolution XPS of Sn 3d.

To further verify the near-surface chemical composition and the oxidation state of element Sn in the as-prepared SnO_2_ products, XPS measurement was then performed, as shown in Figure 3. A survey XPS spectrum of SnO_2_ nanosheet is clearly observed in Figure 3a, indicating the existence of Sn and O elements. For the high resolution of element Sn, two strong peaks centred at 486.8 and 495.2 eV in the XPS spectrum could match with Sn 3d_5/2_ and 3d_3/2_ states, respectively. This clearly indicated that tin was in the Sn (IV) state in the nanosheets sample, which is in good accordance with previously reported SnO_2_ [26].

**Figure 4:**
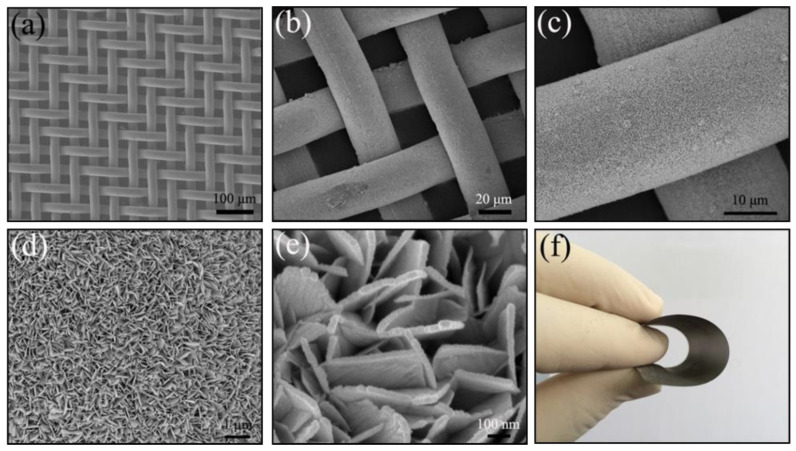
SEM images of (**a**) pristine copper microfibre cloth, where sub-millimetre pores can be clearly observed; (**b**) and (**c**) low magnification images of the copper textile surfaces after SnO_2_ nanosheets layers are grown; (**d**) and (**e**) high-magnification SnO_2_ nanosheets; (**f**) shows a photo of a piece of folded copper textile coated SnO_2_ nanosheets.

FE-SEM and TEM images with different magnifications could provide information about the surface morphology and crystallographic properties of the as-received products. Figure 4a shows a typical SEM image of the uncovered SnO_2_ nanosheets micro-fibre cloth composed of perpendicular and smooth copper micro-fibre, which was interconnected into a textile structure (20 × 20 μm^2^ squared pore) and served as the backbone for the growth of SnO_2_ nanosheets. As shown from Figure 4b, the hydrothermal treatment resulted in a significant morphology change of the copper micro-fibre cloth from a relatively smooth surface to a very rough surface, which indicated that the conductive substrate had been covered with the numerous SnO_2_ nanosheets and formed a CMF@SnO_2_ hybrid material. Based on recorded top-view high magnification SEM images (Figure 4c–e), the outer sheath in the presented 1D architecture consisted of a uniform sheet structure with a smooth surface. Apparent open space between adjacent SnO_2_ nanosheets presented sponge-like porous architecture from the magnified SEM images (Figure 4d,e), which provided a large interfacial area for electrolyte ion diffusion and ensured a short solid-state diffusion length for fast Li-ion insertion/extraction. The direct growth of SnO_2_ nanosheets on the copper micro-fibre network collector enabled good contact and strong binding between SnO_2_ and copper micro-fibre without any binder and carbon black. Due to the high surface energy effect of nanostructured materials, a large number of nanosheets were attached to the outer of the integrated electrode (Supplementary Figure S2), which maybe bring more negative effects on the subsequent electrochemical testing. Hence, the SnO_2_-integrated electrode was purified under ultrasonic treatment. As a result, SnO_2_ nanosheets in situ grown on the CMF were not peeled off even after repeated bending or ultrasonic treatment due to the present interconnected nanoarchitecture. Figure 4f shows the photograph of a copper textile coated with a layer of grey SnO_2_ nanosheets. Notably, similar to micro-fibre cloth, the SnO_2_-integrated electrode with the nanosheets coating could be easily bent without damage to the nanosheets, making them interesting for flexible batteries.

**Figure 5:**
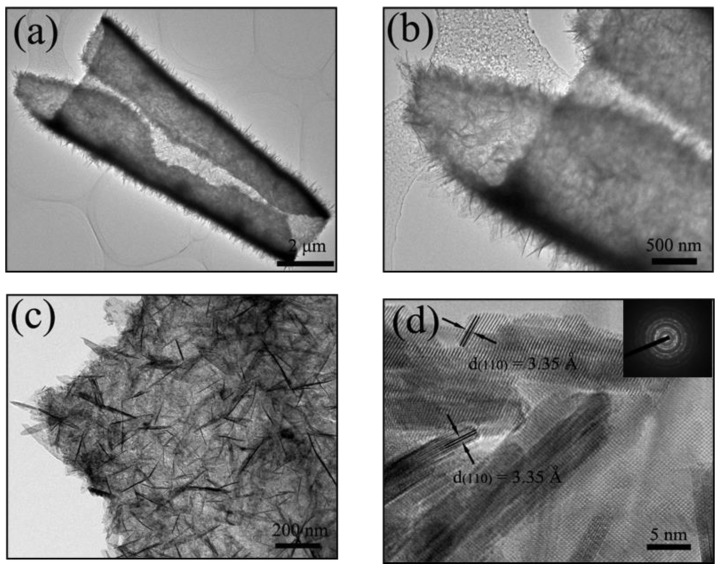
(**a**–**c**) Typical TEM images of the nanosheets SnO_2_; (**d**) HRTEM images of the nanosheets SnO_2_. The inset of d shows that these nanosheets are polycrystalline.

Further microstructure information about SnO_2_ nanosheets building blocks was obtained from transmission electron microscopy (TEM) (Figure 5). As shown in Figure 5a–d, the nanosheets structure could be clearly seen at low magnification. TEM further revealed that the hierarchical SnO_2_ nanoarchitecture was built up of highly porous interconnected nanosheets. The curled geometrical morphology further showed that the presented SnO_2_ electrode materials exhibited significantly improved microstructural flexible performance through nanostructured engineering. The average lateral size and thickness of the nanosheets were found to be approximately 300 and 20 nm, respectively. The morphology and size of the sample obtained from TEM were in good agreement with those observed in the SEM images. Furthermore, Figure 5d shows the HR-TEM image and measured lattice fringes as 3.35 Å, which is consistent with the (111) interplanar distance of the SnO_2_ phase. The inset of Figure 5d is the SAED pattern of a piece of SnO_2_ nanosheets film, which clearly demonstrates the polycrystalline nature of the nanosheets films.

To gain insight into the effect of ammonium fluorides (NH_4_F) on the morphology of SnO_2_ nanostructure, fluoride-dependent experiments were carried out. As shown in Supplementary Figure S3a, SnO_2_ nanoparticles with an irregular shape and size of several nanometres were obtained in the absence of NH_4_F, which confirmed that the NH_4_F was key to the formation of the 2D SnO_2_ nanoarchitecture. When the F/Sn molar ratio was 1, hollow-nanosphere-shaped samples with coarse surfaces could be detected (Supplementary Figure S3b). Once the F/Sn molar ratio was increased to 2, the well-defined 3D flower-like SnO_2_ hollow nanoarchitecture (or nanoflower) constructed by many 2D nanosheets were generated, and their detailed characteristics are described in Supplementary Figure S3c,d. However, the SnO_2_ products could not be found in the Teflon-lined stainless-steel autoclave if the molar ratio was increased to 4.

To prove the efficacy of the as-synthesis unique CMF@SnO_2_ nanosheets architecture as a potential anode material suitable for practical application, we next performed an electrochemical evaluation of this integration electrode without adding any binders (PVDF) and conductive black additives (CB) in half-cells. It is well known that the electrochemical reaction process in the anode can be divided into two steps, as follows:

SnO_2_ + 4Li^+^ + 4e^−^ → Sn + 2Li_2_O (1)

Sn + *x*Li^+^ + *x*e^−^ ↔ Li_x_Sn (0 ≤ *x* ≤ 4.4) (2)

Li^+^ + e^−^ + electrolyte → SEI layer (3)

**Figure 6:**
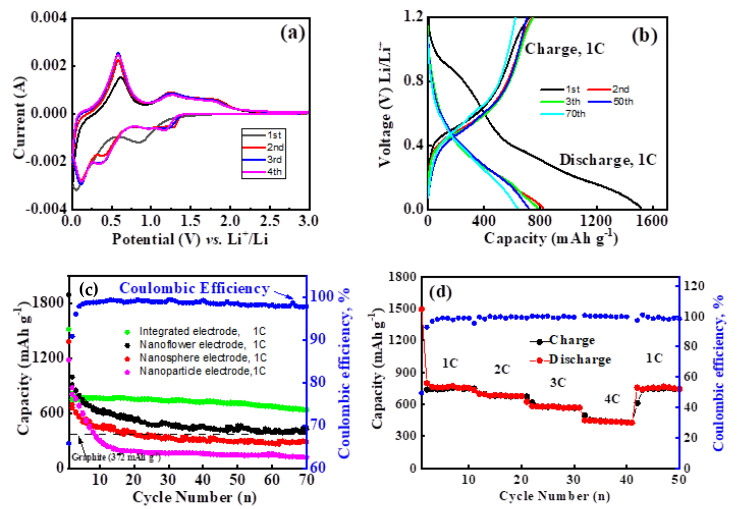
(**a**) Cyclic voltammograms (CVs) of nanosheet-assembled SnO_2_-integrated electrode; (**b**) discharge–charge voltage profile of nanosheet-assembled SnO_2_-integrated electrode between 0.01 and 1.2 V at a rate of 1 C; (**c**) cycling performance and coulombic efficiency (CE) of nanostructured SnO_2_ electrode; (**d**) cycling performance at different rates (1–4 C) of nanosheet-assembled SnO_2_-integrated electrode.

To gain better insight into the electrochemical performance of the novel integration anode, cyclic voltammetry (CV) characteristics of the initial four cyclic were first performed between 0.01 and 3 V, at a scan rate of 0.1 mV s^−1^. As can be seen from Figure 6a, there was a substantial difference between the first and the subsequent cycles. In the first cathodic process, there were significant reduction peaks at 0.8 to 1.0 V, which is related to the conversion of tin dioxide to metallic tin and lithium oxide, and the formation of SEI layer on the electrode surface, as illustrated in Equations (1) and (3) [23,24]. Note that there was a strong reduction peak located at around 0.05 V, which is assigned to the formation of the Li_x_Sn alloys process (Equation (2)). In the subsequent anodic scan, a strong peak at 0.6 V corresponded to the phase transition from Li_x_Sn alloy to metallic Sn, which increased in intensity as the cycle number was increased. This phenomenon could be explained by the activation of the reversible reaction that occurred in the electrode materials [27]. Beyond that, oxidation peaks around 1.3 and 1.9 V were also observed for the SnO_2_-integrated anode materials [23,24,28]. This observation might suggest partial reversibility of the reaction by Equation (1), which is also observed for the nano-sized SnO_2_ and SnO_2_/carbon composites [29,30]. In the following process of CV testing, the peak potentials of anodic and cathodic curves were almost coincident, and the peak intensity changed very slightly, indicating robust cycling stability of SnO_2_-integrated anode.

Furthermore, the galvanostatic charge and discharge measurements of the nanosheet-assembled SnO_2_-integrated electrode were carried out at a high rate of 1 C for 1st, 2nd, 3rd, 50th, and 70th cycles in order to study Li-storage performance. It can be clearly seen that the nanosheet-assembled SnO_2_-integrated electrode showed a similar curve to those of SnO_2_ anode materials and delivered a large initial discharge and charge capacity of 1518.1 and 818.6 mAh g^−1^, respectively. The capacity loss, compared with the first cycle, may be mainly attributed to irreversible side reactions, such as the trapping of some lithium in the lattice, formation of solid–electrolyte interface (SEI) layer, and electrolyte decomposition [23,24,28,29,30]. Nonetheless, perfect reversibility of the capacity was still obtained, and the charge and discharge capacities gradually stabilised in the following 70 cycles.

Cycle stability is another important parameter of the nanosheet-assembled SnO_2_-integrated electrode. Figure 6c displays the cycling performance of the nanosheet-assembled SnO_2_-integrated electrode with a voltage window of 0.01–1.2 V at a current rate of 1 C. It can be seen that the cycling of the nanosheet-assembled SnO_2_-integrated electrode was quite stable, which delivered a high reversible capacity of 637.2 mAh g^−1^ after 70 cycles. It is should be noted that an average Coulombic efficiency of higher than 99% could be obtained up to 70 cycles after the second cycle. In Table S1, we compared the specific capacity of the proposed SnO_2_-integrated electrode with the relevant free-standing anode, including SnO_2_/CNTs, SnO_2_/graphene, and SnO_2_ nanoarchitecture on the metal conductive substrate. Given these results, it is expected that synthesising SnO_2_-integrated anode assembled by nanosheets could promote the electrochemical performance of SnO_2_. Moreover, we also performed the as-synthesised nanoflower, nanosphere, and nanoparticle test as an anode material by the traditional slurry-casting fabrication processes. In comparison, the charge capacity of nanoflower remains 438.5 mAh g^−1^, outperforming that of the theoretical capacity of graphite (372 mAh g^−1^). The nanosphere assembled by nanoparticles had an initial capacity of 1383.3 mAh g^-1^, quickly decreasing to 287.5 mAh g^−1^ after 70 cycles. The nanoparticles without fluorine-doped SnO_2_ showed a relatively low capacity of 123.08 mAh g^−1^ after 70 cycles; in particular, the first 10 cyclings deteriorated sharply with the increasing cycle. It is suggested that such nanosheet-assembled SnO_2_-integrated electrodes could provide more interconnection between the building blocks and a more stable porous structure due to the effective prevention of dense aggregation of the nanosheets. Such a superior Li-storage performance of nanosheet-assembled SnO_2_-integrated electrode should be ascribed to the reasonable structural design on structural engineering to boost synergistic effects. The ultrathin 2D nanoarchitecture blocks provide favourable Li^+^ lateral intercalation lengths and more beneficial transport routes for electrolyte ions, and the interconnected 1D micro-fibre core with outstanding metallic conductivity can offer an efficient electron transport pathway along axial orientation to shorten electron transport. More importantly, the metal’s remarkable flexibility and high tensile strength endow the hybrid integrated electrode with strong bending and stretchability relative to sintered carbon or graphene hosts. Further, for the electrode realised by the traditional slurry-casting fabrication processes, the better cyclability of nanoflower and nanosphere than that of nanoparticle also supports the key role of fluorine-doped strategy [31,32].

To further evaluate the improved electrochemical performance of the SnO_2_-integrated anode, the rate capability at diverse larger current densities was also investigated, which is important for practical applications of LIBs. Benefiting from its unique structure, the nanosheet-assembled SnO_2_-integrated electrode revealed an exceptional cycling response to a continuously varying current rate. As displayed in Figure 6d, the representative specific capacities were about 731.17, 677.55, 567.30, and 423.71 mAh g^−1^ at current rates of 1 C, 2 C, 3 C, and 4 C, respectively. The specific capacity slightly decreased as the current density increased, and it could still be maintained a very stable cycling capacity of above 423.71 mAh g^−1^, corresponding to nearly 100% Coulombic efficiency when the current density was up to 4 C, which was still higher than the theoretical capacity of graphite (372 mAh g^−1^). More importantly, when the current density was adjusted back to the original current density again, the specific capacity of the SnO_2_-integrated electrode still regained the initial reversible value after the high-rate test for the 40 cycles, implying superior stability of the present SnO_2_-integrated electrode.

Overall, using microscopic 2D self-assembled materials combined with a macroscopic 2D metal conductor to prepare integrated electrodes is one of the most promising strategies to increase ion and electron transport kinetics toward present LIBs. According to the above results, the presented SnO_2_-integrated anode showed significantly improved capacity and rate performance. Clearly, the excellent improvement of electrochemical performance of the nanosheets SnO_2_ arrays on the copper micro-fibre electrode can be attributed to two reasons. Firstly, self-supported nanosheets arrays growing directly on a current-collecting substrate represents an attractive nanoarchitecture for LIBs. Such structural feature of thin sheets combined with enriched pores built from the stacking of nanosheets is beneficial to rapid Li^+^ intercalation and diffusion of electrolyte into the inner region of the electrode, high electrode–electrolyte contact area, and good stain accommodation. Moreover, each nanosheet has its own contact with the substrate at the bottom, which can ensure every nanosheet participates in the electrochemical reaction and effectively prevent the aggregation of the SnO_2_ nanosheet. Secondly, the copper grid was selected as an effective substrate because of its high conductivity, 2D planar structure, and larger mechanical strength, compared with carbon materials. Furthermore, the proposed technique also saves the tedious process of mixing active materials with ancillary materials such as carbon black and polymer binders.

## 4. Conclusions

In this study, we presented a cost-effective, scalable, and effective approach to fabricate SnO_2_ nanosheets cluster arrays directly grown on a 2D-interconnected conductive network with robust mechanical flexibility via a facile hydrothermal route. Such integrated electrodes possess a network configuration, which offers more beneficial transport routes for electrolyte ions and guarantees an intimate contact between active and current collectors. As a result, the SnO_2_-integrated electrode with novel nanoarchitecture shows an excellent electrochemical Li-storage performance with a high capacity up to 637.2 mAh g^−1^ at 1 C rate and excellent rate capability of 423.71 mAh g^−1^ at 4 C rate. Such superior cyclic stability and capacity may benefit from the well-designed electrode to boost synergistic effects, which include shortened Li^+^ diffusion distance in the 2D nanoarchitecture blocks, sufficient void space among the nanosheets to reduce volume expansion, and a substrate with superior flexibility and robust tensile strength. The presented strategy provides a new synthetic idea for engineering tin-based energy storage systems with high electrochemical performance and robust mechanical flexibility.

## Data Availability

Not applicable.

## Supplementary Materials

The following are available online, Figure S1: XRD pattern of nanoflower SnO_2_ assembled by nanosheets without CMF, Figure S2: SEM image of the integrated electrode before ultrasonic treatment, Figure S3: SEM image of SnO_2_ samples at 180 °C for 24 h: (a) without NH_4_F; (b) F/Sn = 1; (c,d) F/Sn = 2, Table S1: Comparison of electrochemical properties of nanosheet-assembled SnO_2_-integrated electrode with other reported Sn-based free-standing anode materials.

## Sample Availability

Samples of the compounds are available from the authors.

## References

[1] Zhou G.M., Li F., Cheng H.M. (2014). Progress in flexible lithium batteries and future prospects. Energy Environ. Sci..

[2] Chen D., Lou Z., Jiang K., Shen G.Z. (2018). Device configurations and future prospects of flexible/stretchable lithium-ion batteries. Adv. Funct. Mater..

[3] Liu W., Song M.S., Kong B., Cui Y. (2017). Flexible and stretchable energy storage: Recent advances and future perspectives. Adv. Mater..

[4] Xia J., Zhang X., Yang Y.G., Wang X., Yao J.N. (2021). Electrospinning fabrication of flexible, foldable, and twistable Sb_2_S_3_/TiO_2_/C nanofibre anode for lithium ion batteries. Chem. Eng. J..

[5] Lin X.P., Xue D.Y., Zhao L.Z., Zong F.Y., Duan X.C., Pan X., Zhang J.M., Li Q.H. (2019). In-situ growth of 1T/2H-MoS_2_ on carbon fibre cloth and the modification of SnS_2_ nanoparticles: A three-dimensional heterostructure for high-performance flexible lithium-ion batteries. Chem. Eng. J..

[6] Zhang M., Li L.H., Jian X.L., Zhang S., Shang Y.Y., Xu T.T., Dai S.G., Xu J.M., Kong D.Z., Wang Y. (2021). Free-standing and flexible CNT/(Fe@Si@SiO_2_) composite anodes with kernel-pulp-skin nanostructure for high-performance lithium-ion batteries. J. Alloy. Compd..

[7] Zhang P.C., Cao M.J., Feng Y., Xu J., Yao J.F. (2021). Uniformly growing Co_9_S_8_ nanoparticles on flexible carbon foam as a free-standing anode for lithium-ion storage devices carbon foam as a free-standing anode for lithium-ion storage devices. Carbon.

[8] Zhou F.G., Han S.C., Qian Q.R., Zhu Y.F. (2019). 3D printing of free-standing and flexible nitrogen doped graphene/polyaniline electrode for electrochemical energy storage. Chem. Phys. Lett..

[9] Hao Y., Wang C. (2020). Free-standing reduced graphene oxide/carbon nanotube paper for flexible sodium-ion battery applications. Molecules.

[10] Shao J., Yang Y., Zhang X., Shen L., Bao N. (2021). 3D yolk–yhell ytructured Si/void/rGO free-standing electrode for lithium-ion battery. Materials.

[11] Li X., Bai Y., Wang M., Wang G., Ma Y., Huang Y., Zheng J. (2019). Dual carbonaceous materials synergetic protection silicon as a high-performance free-standing anode for lithium-ion battery. Nanomaterials.

[12] Huang Y., Li Y.W., Huang R.S., Ji J.C., Yao J.H., Xiao S.H. (2021). One-pot hydrothermal synthesis of N-rGO supported Fe_2_O_3_ nanoparticles as a superior anode material for lithium-ion batteries. Solid State Ionics.

[13] Li B.Q., Zhao W., Yang Z., Zhang C., Dang F., Liu Y.L., Jin F., Chen X. (2020). A carbon-doped anatase TiO_2_-Based flexible silicon anode with high-performance and stability for flexible lithium-ion battery. J. Power Sources.

[14] Li N., Chen Z.P., Ren W.C., Li F., Cheng H.M. (2021). Flexible graphene-based lithium ion batteries with ultrafast charge and discharge rates. Proc. Natl. Acad. Sci. USA.

[15] Liu S.H., Wang Z.Y., Yu C., Wu H.B., Wang G., Dong Q., Qiu J.S., Eychmüller A., Lou X.W. (2013). A Flexible TiO_2_(B)-based battery electrode with superior power rate and ultralong cycle life. Adv. Mater..

[16] Dai L., Zhong X., Zou J., Fu B., Su Y., Ren C., Wang J., Zhong G. (2021). Highly ordered SnO_2_ nanopillar array as binder-free anodes for long-life and high-rate Li-ion batteries. Nanomaterials.

[17] Chen S., Wang M., Ye J., Cai J., Ma Y., Zhou H., Qi L. (2013). Kinetics-controlled growth of aligned mesocrystalline SnO_2_ nanorod arrays for lithium-ion batteries with superior rate performance. Nano Res..

[18] Ding Y., Zhou P., Han T., Liu J. (2020). Environmentally friendly and cost-effective synthesis of carbonaceous particles for preparing hollow SnO_2_ nanospheres and their bifunctional Li-storage and gas-sensing properties. Crystals.

[19] Tran Q.N., Kim I.T., Park S., Choi H.W., Park S.J. (2020). SnO_2_ Nanoflower–nanocrystalline cellulose composites as anode materials for lithium-ion batteries. Materials.

[20] Park M.S., Wang G.X., Kang Y.M., Wexler D., Dou S.X., Liu H.K. (2007). Preparation and electrochemical properties of SnO_2_ nanowires for application in lithium-ion batteries. Angew. Chem. Int. Ed..

[21] Lou X.W., Li C.M., Archer L.A. (2009). Designed synthesis of coaxial SnO_2_@carbon hollow nanospheres for highly reversible lithium storage. Adv. Mater..

[22] Zhou X., Yin Y.X., Wan L.J., Guo Y.G. (2012). A robust composite of SnO_2_ hollow nanospheres enwrapped by graphene as a high-capacity anode material for lithium-ion batteries. J. Mater. Chem..

[23] Li J., Zhao Y., Wang N., Guan L. (2011). A high performance carrier for SnO_2_ nanoparticles used in lithium ion battery. Chem. Commun..

[24] Wang X.Y., Zhou X.F., Yao K., Zhang J.G., Liu Z.P. (2011). A SnO_2_/graphene composite as a high stability electrode for lithium ion batteries. Carbon.

[25] Kim W.S., Hwa Y., Jeun J.H., Sohn H.J., Hong S.H. (2013). Synthesis of SnO_2_ nano hollow spheres and their size effects in lithium ion battery anode application. J. Power Sources.

[26] Ambalkar A.A., Panmand R.R., Kawade U.V., Sethi Y.A., Naik S.D., Kulkarni M.V., Adhyapak P.V., Kale B.B. (2020). Facile synthesis of SnO_2_@carbon nanocomposites for lithium-ion batteries. New J. Chem..

[27] Nguyen T.P., Kim I.T. (2020). Self-assembled few-layered MoS_2_ on SnO_2_ anode for enhancing lithium-ion storage. Nanomaterials.

[28] Reddy M.V., Andreea L.Y.T., Ling A.Y., Hwee J.N.C., Lin C.A., Admas S., Loh K.P., Mathe M.K., Ozoemena K.I., Chowdari B.V.R. (2013). Effect of preparation temperature and cycling voltage range on molten salt method prepared SnO_2_. Electrochim. Acta.

[29] Xu H., Chen J., Wang D., Sun Z.M., Zhang P.G., Zhang Y., Guo X. (2017). Hierarchically porous carbon-coated SnO_2_@graphene foams as anodes for lithium ion storage. Carbon.

[30] Han F., Li W.C., Li M.R., Lu A.H. (2012). Fabrication of superior-performance SnO_2_@C composites for lithium-ion anodes using tubular mesoporous carbon with thin carbon walls and high pore volume. J. Mater. Chem..

[31] Kwon C.W., Campet G., Portier J., Poquet A., Fournes L., Labrugere C., Jousseaume B., Toupance T., Choy J.H., Subramanian M.A. (2001). A new single molecular precursor route to fluorine-doped nanocrystalline tin oxide anodes for lithium batteries. J. Inorg. Mater..

[32] Wang H.K., Fu F., Zhang F.H., Wang H.E., Kershaw S.V., Xu J.Q., Sun S.G., Rogach A.L. (2012). Hydrothermal synthesis of hierarchical SnO_2_ microspheres for gas sensing and lithium-ion batteries applications: Fluoride-mediated formation of solid and hollow structures. J. Mater. Chem..

