# Supplementary material for: A Nanosheet-Assembled SnO2-Integrated Anode
Source: Molecules. 2021 Oct 10;26(20):6108. doi: 10.3390/molecules26206108 (PMC8538835; doi:10.3390/molecules26206108)
Supplement: Supplementary file 1 [file molecules-26-06108-s001.zip › molecules-1344966-supplementary.pdf]

molecules

Supplementary Material

Nanosheets-Assembled Integrated SnO_2 Anode

Xiaoli Wang [1], Xinyu Zhao [2,*] and Yin Wang [2,*]

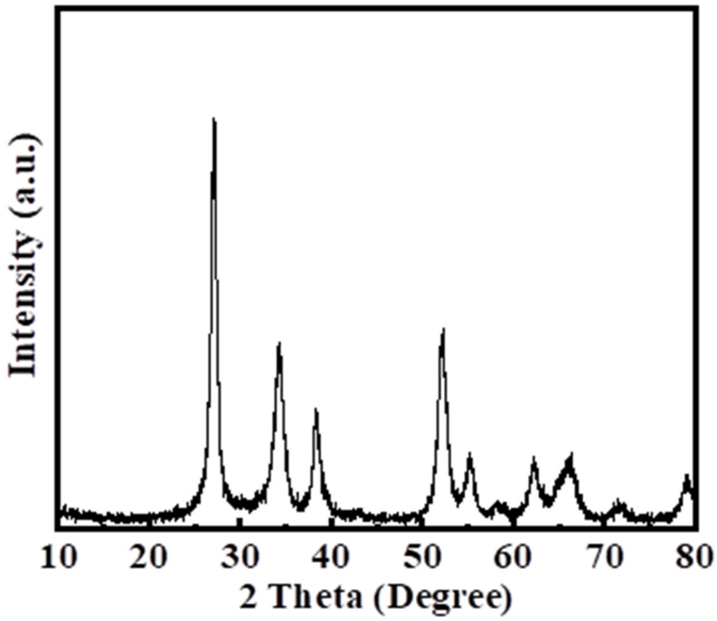

Figure S1. XRD pattern of nanoflower SnO_2 assembled by nanosheets without CMF.

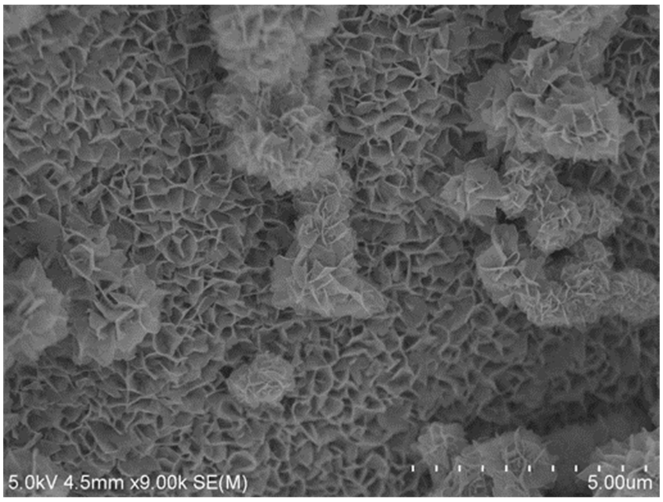

Figure S2. SEM image of integrated electrode before ultrasonic treatment.

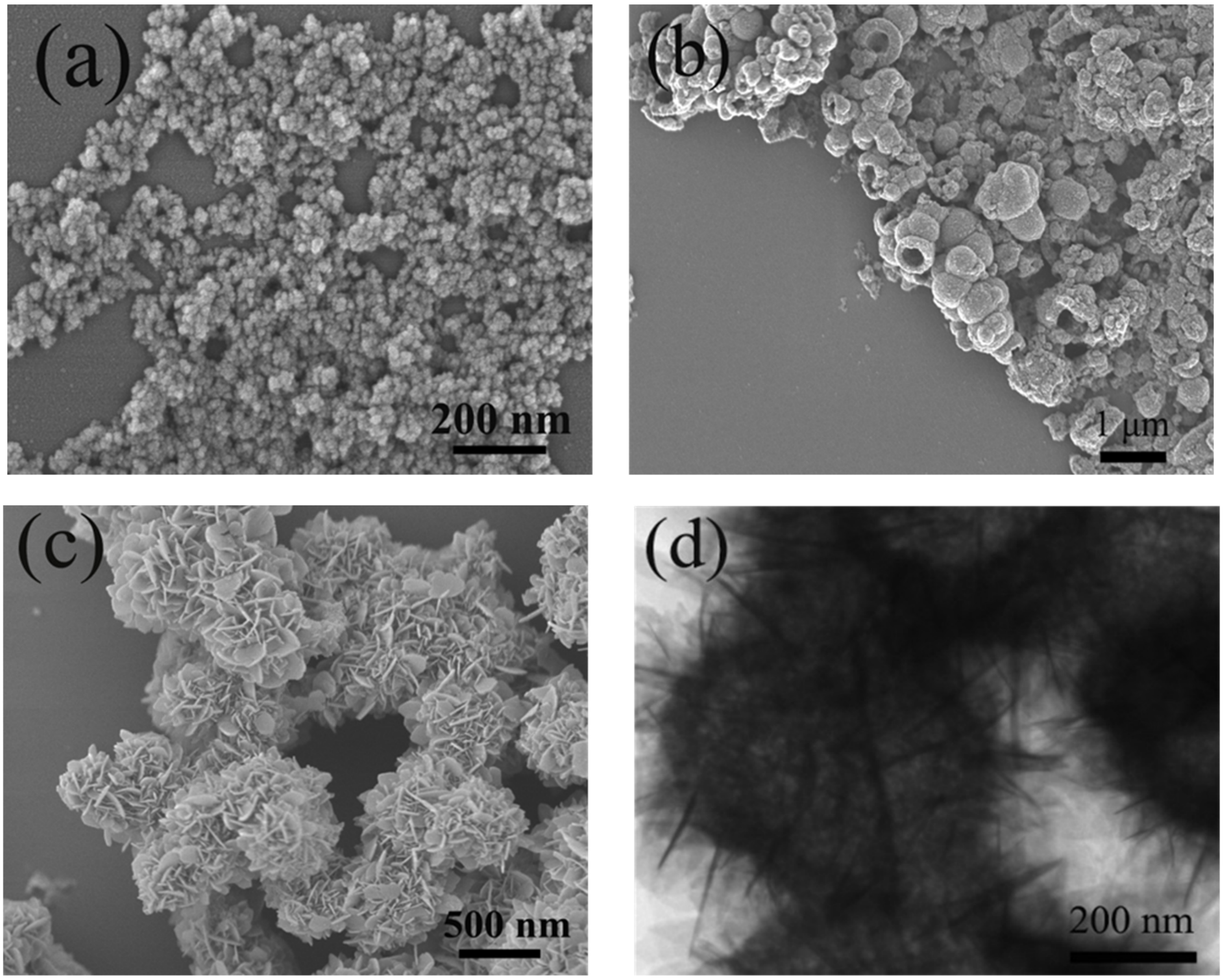

Figure S3. SEM image of SnO_2 samples at 180 °C for 24 h (**a**) without NH_4F; (**b**) F/Sn = 1; (**c**)–(**d**)F/Sn = 2.

Table S1. Comparison of electrochemical properties of nanosheets-assembled SnO_2 integration electrode with other reported Sn-based free-standing anode materials.

Anode Material	Specific Capacity	Current Density	Cycles	Ref.
SWCNT/SnO_2	454 mAh g^{-1}	25 mA g^{-1}	100	[1]
SnO_2 films	524 mAh g^{-1}	100 mA g^{-1}	50	[2]
SnO_2 nanowire	510 mAh g^{-1}	1 C	50	[3]
ZnO/SnO_2/MWCNT	487 mAh g^{-1}	0.2 C	100	[4]
Graphene/Co-doped SnO_2	549.6 mAh g^{-1}	100 mA g^{-1}	100	[5]
SnO_2/CNT	1.23 mAh cm^{-2}	250 mA g^{-1}	20	[6]
Double-layer SnO_2 nanosheets	550 mAh g^{-1}	200 mA g^{-1}	30	[7]
SnO_x film	396.1 mAh g^{-1}	44 μA cm^{-2}	50	[8]
SnO_2 nanorod arrays	580 mAh g^{-1}	0.1 C	100	[9]
SnO_2 nanotube	525 mAh g^{-1}	0.05 mA cm^{-2}	80	[10]
SnO_2 integrated electrode	637.2 mAh g^{-1}	1 C	70	This work

References

1. Noerochim, L.; Wang, J.Z.; Chou, S.L.; Wexler, D.; Liu, H.K. Free-standing single-walled carbon nanotube/SnO_2 anode paper for flexible lithium-ion batteries. *Carbon* **2012**, *50*, 1289–1297.

2. Wang, H.J.; Wang, J.M.; Fang, W.B.; Wan, H.; Liu, L.; Lian, H.Q.; Shao, H.B.; Chen, W.X.; Zhang, J.Q.; Cao, C.N. Structural and electrochemical properties of a porous nanostructured SnO_2 film electrode for lithium-ion batteries. *Electrochem. Commun.* **2010**, *12*, 194–197.
3. Ko, Y.D.; Kang, J.G.; Park, J.G.; Lee, S.J.; Kim, D.W. Self-supported SnO_2 nanowire electrodes for high-power lithium-ion batteries. *Nanotechnology* **2009**, *20*, 455701–455706.
4. Kose, H.; Dombaycioglu, S.; Aydin, A.O.; Akbulut, H. Production and characterization of free-standing ZnO/SnO_2/MWCNT ternary nanocomposite Li-ion battery anode. *Int. J. Hydrogen Energy* **2016**, *41*, 9924–9932.
5. Zhang, X.Q.; Huang, X.X.; Zhang, X.D.; Xia, L.; Zhong, B.; Zhang, T.; Wen, G.W. Flexible carbonized cotton covered by graphene/Co-doped SnO_2 as free-standing and binder-free anode material for lithium-ions batteries. *Electrochim. Acta* **2016**, *222*, 518–527.
6. Abnavi, A.; Faramarzi, M.S.; Sanaee, T.; Ghasemi, S. SnO_2 nanowires on carbon nanotube film as a high performance anode material for flexible Li-ion batteries. *J. Nanostruct 8*, *2018*, 288–293.
7. Zhang, L.; Wu, H.B.; Lou, X.W. Growth of SnO_2 nanosheet arrays on various conductive substrates as integrated electrodes for lithium-ion batteries. *Mater. Horiz. 1*, **2014**, 133–138.
8. Ma, Y.; Zhang, X.; Liu, W.; Wei, Y.; Fu, Z.; Li, J.; Zhang, X.; Peng, J.; Yan, Y. Stoichiometry dependence of physical and electrochemical properties of the SnOx film anodes deposited by pulse DC magnetron sputtering. *Materials* **2021**, *14*, 1803–1823.
9. Liu, J.P.; Li, Y.Y.; Huang, X.T.; Ding, R.M.; Hu, Y.Y.; Jiang, J.; Liao, L. Direct growth of SnO_2 nanorod array electrodes for lithium-ion batteries. *J. Mater. Chem.* **2009**, *19*, 1859–1864.
10. Wang, Y.; Lee, J.Y.; Zeng, H.C. Polycrystalline SnO_2 nanotubes prepared via infiltration casting of nanocrystallites and their electrochemical application. *Chem. Mater.* **2005**, *17*, 3899–3903.
